# CXCR3-dependent recruitment and CCR6-mediated positioning of Th-17 cells in the inflamed liver

**DOI:** 10.1016/j.jhep.2012.07.008

**Published:** 2012-11

**Authors:** Ye Htun Oo, Vanessa Banz, Dean Kavanagh, Evaggelia Liaskou, David R. Withers, Elizabeth Humphreys, Gary M. Reynolds, Laura Lee-Turner, Neena Kalia, Stefan G. Hubscher, Paul Klenerman, Bertus Eksteen, David H. Adams

**Affiliations:** 1 Centre for Liver Research & NIHR Biomedical Research Unit in Liver Disease, University of Birmingham, Birmingham, United Kingdom; 2 Centre for Cardiovascular Sciences, University of Birmingham, Birmingham, United Kingdom; 3 MRC Centre for Immune Regulation, University of Birmingham, Birmingham, United Kingdom; 4 Department of Visceral Surgery, Inselspital, University of Berne, Switzerland; 5 Peter Medawar Building for Pathogen Research, Nuffield Department of Clinical Medicine, University of Oxford, Oxford, United Kingdom

**Keywords:** Th17, interleukin-17 secreting CD4 T helper cells, Tc17, interleukin-17 secreting CD8 T helper cells, LIL, liver infiltrating lymphocytes, HSEC, hepatic sinusoidal endothelial cell, BEC, biliary epithelial cells, RORC, retinoic acid-related orphan receptor c, AIH, autoimmune hepatitis, HCV, chronic hepatitis C, PBC, primary biliary cirrhosis, ALD, alcoholic liver disease, NANB, non-A non-B acute hepatitis, NASH, non-alcoholic steato-hepatitis, NL, normal liver, CCL_4_, carbon tetrachloride, ConA, concanavalin A, TNF-α, tumour necrosis factor-α, IFN-γ, interferon gamma, CFSE, carboxyfluorescein succinimidyl ester, Interleukin-17, Hepatitis, Th17 cells, Tc17 cells, Liver, Bile ducts, Chemokine receptor, Chemokine, Concanavalin A

## Abstract

**Background & Aims:**

IL-17 secreting CD4 (Th17) and CD8 (Tc17) T cells have been implicated in immune-mediated liver diseases, but the molecular basis for their recruitment and positioning within the liver is unknown.

**Methods:**

The phenotype and migratory behaviour of human liver-derived Th17 and Tc17 cells were investigated by flow cytometry and chemotaxis and flow-based adhesion assays. The recruitment of murine Th17 cells to the liver was studied *in vivo* using intra-vital microscopy.

**Results:**

IL-17^+^ T cells comprised 1–3% of the T cell infiltrate in inflammatory liver diseases and included both CD4 (Th17) and CD8 (Tc17) cells. They expressed RORC and the IL-23 receptor and included subsets that secreted IL-22 and interferon-γ. Th17 and Tc17 cells expressed high levels of CXCR3 and CCR6, Tc17 cells also expressed CXCR6. Binding to human sinusoidal endothelium from flow was dependent on β1 and β2 integrins, CXCR3, and, in the case of Th17 cells, VAP-1. Th17 recruitment via sinusoids in mice with liver inflammation was reduced by treatment with antibodies against CXCR3 ligands, confirming the role of CXCR3 in Th17 recruitment *in vivo*. In human liver, IL-17^+^ cells were detected in portal infiltrates close to inflamed bile ducts expressing the CCR6 ligand CCL20. Cytokine-treated human cholangiocytes secreted CCL20 and induced CCR6-dependent migration of Th17 cells suggesting that local cholangiocyte chemokine secretion localises Th17 cells to bile ducts.

**Conclusions:**

CXCR3 promotes recruitment of Th17 cells from the blood into the liver in both human and murine liver injury. Their subsequent positioning near bile ducts is dependent on cholangiocyte-secreted CCL20.

## Introduction

Th17 are a distinct subset of CD4 effector cells [1], [2] that develop under control of the nuclear receptor RORc in humans (RORγt in mice) [3], [4], in response to antigen priming in an environment rich in IL-6 and TGF-β [5], [6]. Th17 cells secrete cytokines IL-17A, IL-17F, IL-22, TNF-α, and IFN-γ [2] and provide protection against pathogens at mucosal sites [7], [8]. Stimulation with IL-6, IL-21 or IL-1β and TGF-β increases expression of the IL-23 receptor [9] through which IL-23 stabilises the Th17 phenotype [2], [9], [10]. Both Th1 and Th17 cells have been implicated in inflammation and autoimmunity [11] and IL-23 shares the p40 subunit with the classical Th1 cytokine IL-12 [12]. In some antigen-driven models of autoimmunity, diseases can be transferred by Th17 cells alone although it is not yet certain how effective targeting IL-17 will prove in clinical disease [13], [14].

Th17 cells play a role in chronic inflammatory liver diseases [2]. Numbers of circulating and intra-hepatic Th17 cells correlate with viral load and histological inflammation in chronic viral hepatitis [15], [16] and the frequency of intra-hepatic Th17 cells correlates with disease severity in alcoholic liver disease [17]. Pro-inflammatory Th17 cells accumulate in hepatocellular carcinoma and promote disease progression [18]. In primary biliary cirrhosis, Th17 cells are detected in the portal tracts near damaged bile ducts [19]. Conversely, the Th17 cytokine IL-22 is hepato-protective in acute inflammation and ameliorates alcoholic liver injury [20], [21]. We have recently reported high frequencies of Tc17 in human HCV-infected livers that correlate with control of disease progression [22].

Although both Th17 and Tc17 are detected in chronic hepatitis, little is known about the molecular basis of their recruitment and subsequent positioning within the liver. We had previously shown that both effector and regulatory T cells [23], [24] used the chemokine receptor CXCR3 and its ligands to enter the liver via sinusoidal endothelium [23], [25]. In the present study, we show that CXCR3 is also critical for Th17 recruitment from the blood into the inflamed liver and that CCR6 is involved in subsequent positioning at epithelial interfaces.

## Materials and methods

Human blood and liver tissue were collected with informed consent at liver transplantation. C57BL/6 mice were obtained from existing colonies at the University of Birmingham.

Isolation of peripheral blood lymphocytes (PBL), liver-infiltrating lymphocytes (LIL), and biliary epithelial cells (BEC) was carried out using published methods described in Supplementary Materials and methods [23], [24], [26].

### Human and murine Th17 cells isolation

IL-17 cells were isolated using IL-17 Enrichment & Detection Kit (Miltenyi; purity >95%). Tc17 and Th17 cells were generated from CD4^+^/CD8^+^ cells, murine splenocytes or human PBL stimulated with anti-CD3/CD28 beads in Iscove’s Modified Dulbecco’s Medium supplemented with Th17 polarising cytokines and anti-cytokine mAbs (see Supplementary Materials and methods).

### CCL20 measurement

CCL20 chemokine was measured in human BEC supernatants by ELISA (Supplementary Materials and methods) and *CCL20* and *IL-17RA* mRNA was extracted from BEC and quantified by RT-PCR (Supplementary Materials and methods).

### Immunohistochemistry and confocal microscopy

Human paraffin liver tissues were stained for immunohistochemistry and images captured with a Zeiss microscope (Supplementary Materials and methods) [23].

### Flow cytometry

Freshly isolated LIL from human and murine livers were stained for surface and chemokine receptors before *in vitro* stimulation followed by intracellular cytokine and transcription factors staining (Supplementary Materials and methods).

### Th17 chemotaxis

Primary BEC cultures were stimulated with IL-17A or medium alone for 24 h, supernatants collected and placed in the bottom wells of 5-μ pore transwells (Corning) with Th17 cells in the upper chamber in the presence or absence of blocking antibodies (Supplementary Materials and methods).

### Flow-based adhesion assays

Recruitment of Th17/Tc17 by the hepatic endothelium *in vitro* was studied using a flow-based adhesion assay in which HSEC were cultured in micro-capillaries, stimulated for 24 h with TNF-α & IFN-γ prior to perfusion of cells at a wall shear stress of 0.05 Pa. Adherent cells were visualised by phase contrast microscopy (10× objective) (Supplementary Materials and methods).

### Murine liver injury models and intra-vital microscopy

*In vitro* generated Th17 cells were labelled with 5 μM CFSE (Molecular Probes, Invitrogen) and 5 × 10^6^ cells injected into mice with either ConA hepatitis or CCL_4_-induced liver injury. Th17 interactions with hepatic vessels were imaged using intravital microscopy and a Sensicam CCD camera (Supplementary Materials and methods).

### Statistical analysis

Data were analysed with Student’s *t*-test when comparing numerical variables between two groups. One-way ANOVA analysis followed by Newman–Keul *post hoc* analysis or Bonferroni correction was used for comparisons between more than two groups. Statistical analyses were performed using GraphPad Prism software. A value of *p *<0.05 was considered statistically significant. Data are presented as mean ± SEM.

## Results

### Distribution, frequency and subsets of IL-17 cells in human liver

**Fig. 1:**
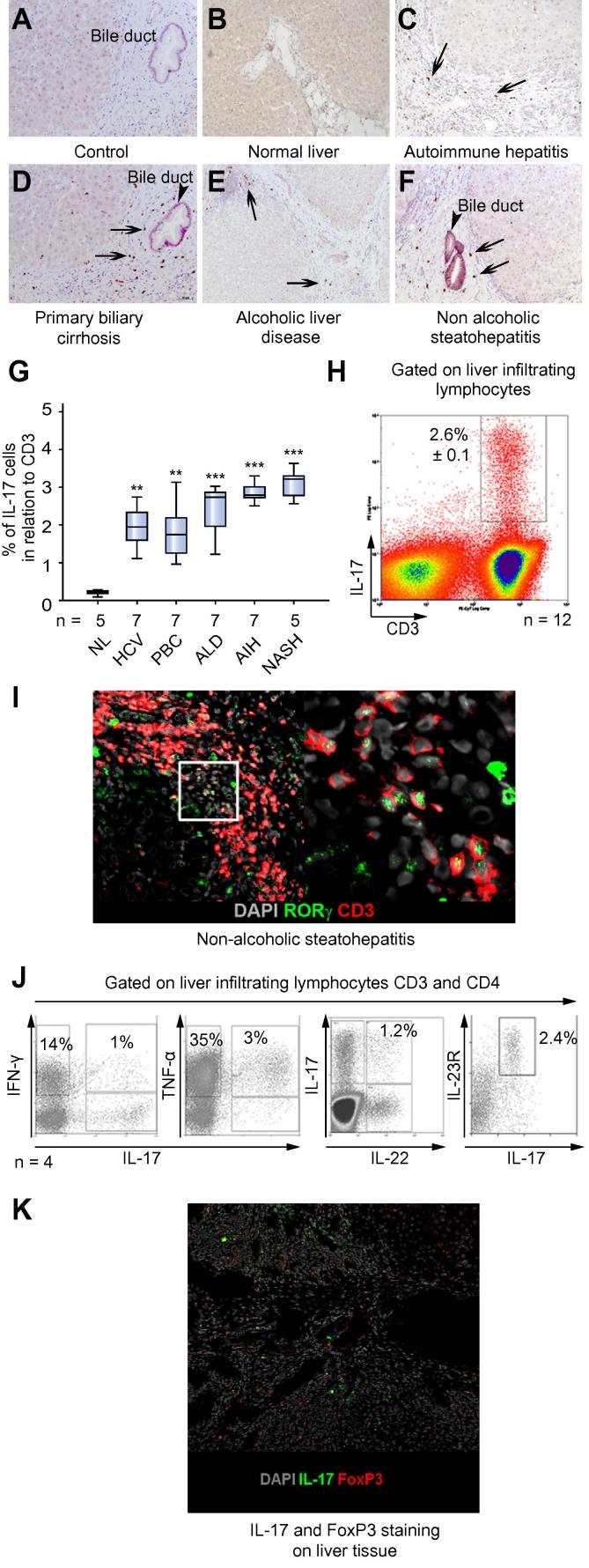
**Distribution and frequency of IL-17 producing cells in human liver**. (A–F) IL-17^+^ cells (arrows) in human livers are shown. (A) Control antibody staining and IL-17A on (B) normal and (C–F) human livers from different diseases. Bile ducts are indicated by arrow-heads. (G) Proportion of CD3 T cells staining for IL-17 for different liver diseases analysed by immunohistochemistry counting (one-way ANOVA test; ^∗∗∗^*p *⩽ 0.0001; ^∗∗^*p *⩽ 0.001). (H) IL-17 expressing CD3 lymphocytes in human LIL (representative histogram of patient with autoimmune hepatitis, mean ± SEM; N = 12). (I) Confocal microscopy staining of RORc^+^ CD3^+^ cells in portal tracts; CD3 (red/TritC); RORc (green/FITC); nucleus (DAPI), magnification 10× (one representative sample of non-alcoholic steatohepatitis is shown; N = 4). The right hand panel shows an enlarged picture of marked area, magnification 40×. (J and Table 1) Percentage co-expression of different cytokines on liver infiltrating Th17 cells is shown. Liver infiltrating lymphocytes were stimulated for 5 h with PMA and ionomycin and brefeldin A Golgi block was applied for the last 2 h before staining for intracellular cytokines. LITh17 (liver infiltrating Th17) cells express the IL-23 receptor (liver infiltrating lymphocytes were gated on CD3 and CD4; representative flow plots of an ALD patient are shown). Four diseased livers were studied (2× AIH, PBC, and ALD). (K) Confocal images of FoxP3^+^ regulatory T cells and IL17 cells in a patient with PBC, FoxP3 (red/TritC), IL-17 (green/FITC), nucleus (DAPI). An autoimmune hepatitis liver is shown, magnification 10×. Data is representative of 4 samples. [This figure appears in colour on the web.]

Immunohistochemistry revealed that the normal human liver contained very few IL-17 cells (Fig. 1B and G) whereas numbers of intra-hepatic IL-17^+^ cells increased in all chronic liver diseases studied (Fig. 1C–F, and G). IL-17^+^ cells were detected in portal infiltrates (Fig. 1F) with preferential localization around bile ducts, particularly in PBC (Fig. 1D).

**Fig. 2:**
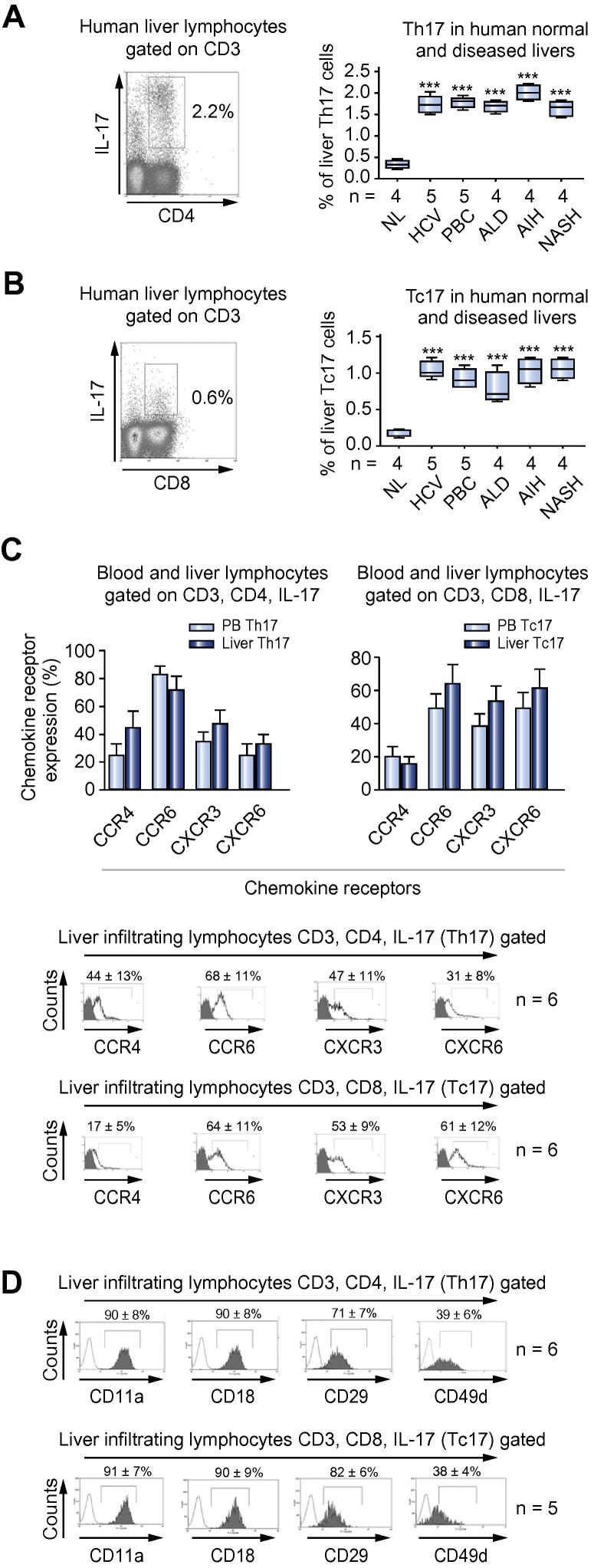
**Frequency and chemokine receptors expression on human intra-hepatic Th17 and Tc17 cells.** (A) Liver infiltrating Th17 and (B) Tc17. One representative FACS blot of cells isolated from an autoimmune hepatitis liver is shown. Freshly isolated liver-infiltrating lymphocytes were gated on forward and side scatter and then re-gated on CD3. Percentage expression of Th17 and Tc17 in normal and diseased livers analysed by flow cytometry is shown (one-way ANOVA with Bonferroni *post hoc* correction; ^∗∗∗^*p *⩽0.0001). (C) Percentage chemokine receptor expression on peripheral blood (N = 5) and liver-infiltrating Th17 and Tc17 cells (N = 6) is shown. Percentage expression is shown as mean ± SD. Bottom panel, flow cytometry overlay of the expression of liver infiltrating Th17 and Tc17 chemokine receptors (N = 6). (D) β1 and β2 integrin expression on Th17 and Tc17 by flow cytometry (representative overlay plot N = 6 for Th17; N = 5 for Tc17; percentage expression is shown as mean ± SD). [This figure appears in colour on the web.]

IL-17^+^ cells comprised around 2–3% of the CD3 T cell infiltrate in liver disease, as defined by frequencies generated by immunohistochemical analysis (Fig. 1G) and by calculating the frequencies of CD3^+^ IL-17 secreting cells, in cells freshly isolated from liver tissue by flow cytometry (Fig. 1H). IL-17^+^ cells in tissue analysed using confocal microscopy revealed both CD3 and RORc expressing cells (Fig. 1I). These CD3 IL17 cells are composed of both Th17 (CD4 IL-17^+^) and Tc17 (CD8 IL-17^+^) cells (Fig. 2A and B). A proportion of Th17 also secreted IL-22. A CD4^+^IL-22 producing population was detected consistent with previous reports on Th22 [28] (Fig. 1J and Table 1). Th17 cells included poly-secreting populations that expressed TNF-α and IFN-γ in addition to IL-17 (Fig. 1J and Table 1). Because T regulatory cells have been shown to secrete IL-17 at sites of inflammation, we looked for co-localisation of IL-17 and FoxP3. Although both IL-17^+^ and FoxP3^+^ cells were detected, no co-expression was observed, suggesting that regulatory T cells in the inflamed liver do not secrete IL-17 (Fig. 1K).

**Table 1:** **Intracellular cytokines co-expression in different diseased human liver infiltrating Th17 lymphocytes**.

### Frequency, phenotype, and chemokine receptors expression of human intra-hepatic Th17/Tc17 cells

Lymphocytes were freshly isolated from explanted human livers and analysed. Th17 and Tc17 cells were present in all liver diseases studied (Fig. 2A and B). Tc17 were also present in inflamed livers at slightly lower frequencies than Th17 (Fig. 2A and B). Very few liver-infiltrating Th17 and Tc17 cells were present in the normal liver. There was no significant difference between diseases (Fig. 2A and B).

**Supplementary Fig. 1:**
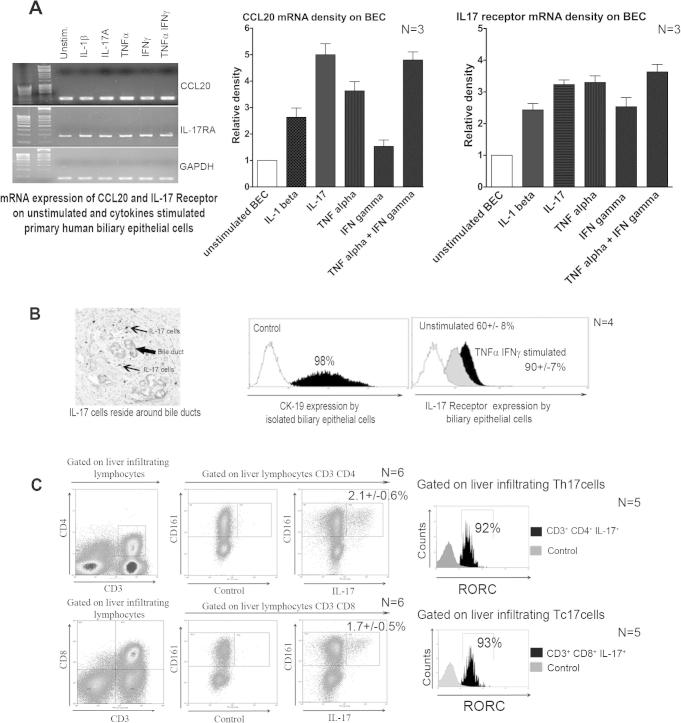


Both Th17 and Tc17 expressed high levels of RORC and were found within the CD161^high^ population, as previously reported (Supplementary Fig. 1C) [22]. Human liver-infiltrating Th17 expressed high levels of chemokine receptors, CCR6 68 ± 11% (mean ± SD), CXCR3 47 ± 11%, and CCR4 44 ± 13%, irrespective of the cause of liver disease (Fig. 2C). We have recently reported that intra-hepatic CD161^+^Tc17 express high levels of CXCR6 [22] and this was confirmed with CXCR6 being expressed on 61 ± 12% of Tc17. However, CXCR6 levels were lower on intra-hepatic Th17 cells. CCR6 and CXCR3 were expressed at similar levels on intra-hepatic Tc17 cells (CCR6 64 ± 11%, CXCR3 53 ± 9%; Fig. 2C). Th17 and Tc17 cells expressed high levels of β1 and β2 integrins (Fig. 2D).

### Presence of IL-17^+^ cells around bile ducts and expression of the IL-17 receptor on BEC

IL-17 cells were found close to bile ducts within inflamed portal tracts (Fig. 1D and Supplementary Fig. 1B), as previously reported [19]. We investigated the expression of the IL-17 receptor on BEC to determine whether these cells had the potential to respond to locally secreted IL-17 by Th17/Tc17. Human BEC expressed cell surface IL17RA (Supplementary Fig. 1B) and *IL-17RA* mRNA was detected on BEC and increased after cytokine treatment (Supplementary Fig. 1A).

### Human BEC express and secrete CCL20

**Fig. 3:**
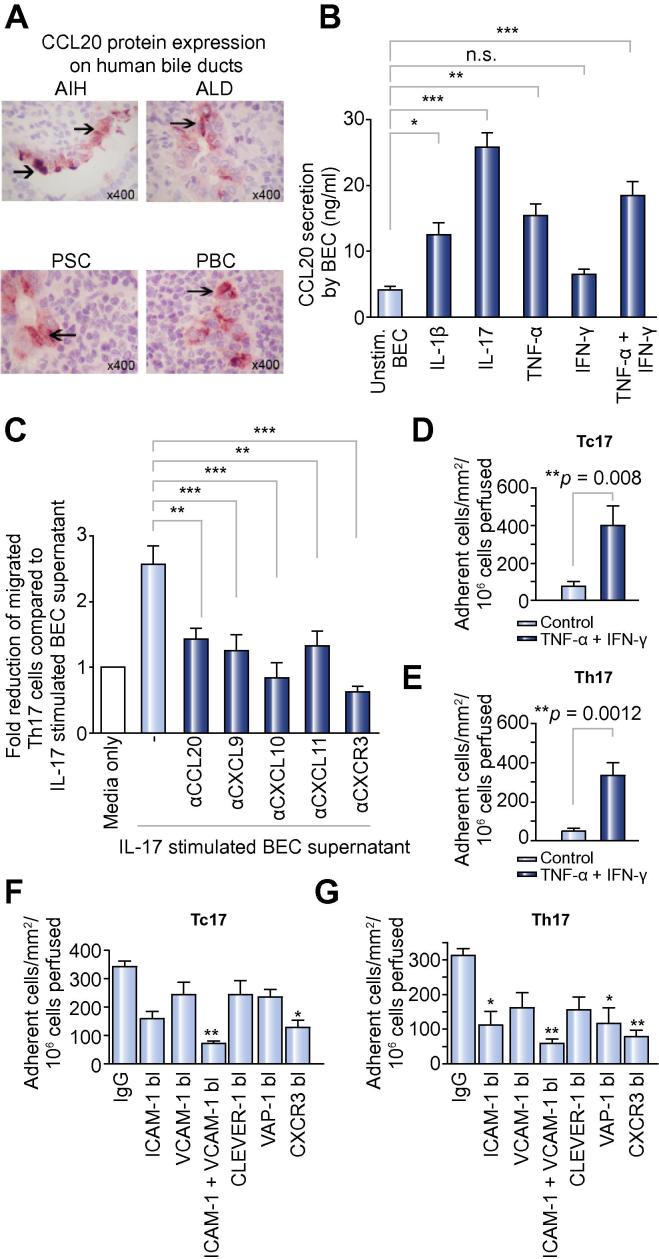
**CCR6-dependent positioning around bile ducts and CXCR3-mediated recruitment of Th17 and Tc17**. (A) CCL20 staining (arrow heads) of bile ducts on paraffin-embedded diseased human liver sections (AIH, autoimmune hepatitis; ALD, alcoholic liver disease; PSC, primary sclerosing cholangitis; PBC, primary biliary cirrhosis). (B) CCL20 secretion in human BEC supernatant determined by sandwich ELISA (one-way ANOVA test; ^∗∗∗^*p *⩽0.0001; ^∗∗^*p *⩽0.001; ^∗^*p *⩽0.01; n.s., not significant) comparing medium alone to individual cytokine-stimulated BEC supernatant; N = 8 different diseased BEC. (C) Culture supernatants of IL-17-stimulated BEC were placed in the lower chambers for transwell assay and migration of Th17 quantified. Antibodies were used to deplete CCL20 (CCR6 ligand) and CXCL9–11 (CXCR3 ligands) in the BEC conditioned medium (N = 8, one-way ANOVA with Bonferroni correction, ^∗∗^*p *<0.001; ^∗∗∗^*p *<0.0001 comparing IL-17-stimulated BEC supernatant and supernatant after chemokine depletion/blockade or anti-CXCR3 treatment of Th17 cells). (D and E) Tc17 and Th17 lymphocyte adhesion to hepatic sinusoidal endothelial cells from flow. HSEC were stimulated with TNF-α (10 ng/ml) and IFN-γ (10 ng/ml) for 24 h prior to perfusion of (D) Tc17 and (E) Th17 lymphocytes at a shear stress of 0.05 Pa. Adhesion was classified as rolling, static adhesion or migration, which were combined to give the total number of adherent cells ± SEM from N = 4 different Tc17 and Th17 cell preparations (F and G). Adhesion of Th17 and Tc17 cells (total numbers) on TNF-α/IFN-γ-stimulated HSEC was reduced by function-blocking antibodies against ICAM-1 and VCAM-1, CLEVER-1, VAP-1, on HSEC or anti-CXCR3 block on Tc17/Th17 cells compared to adhesion observed with a control antibody (F and G, Supplementary Fig. 2). ^∗^*p *<0.01, ^∗∗^*p *<0.001 by one-way ANOVA with Bonferroni correction. [This figure appears in colour on the web.]

The CXCR3 ligands CXCL9–11 are known to be expressed by hepatocytes, cholangiocytes, and stellate cells in liver disease [25], [29] but less is known about the CCR6 ligand CCL20. We detected CCL20 on intrahepatic bile ducts in inflamed human livers (Fig. 3A) but not on other liver cells. To elucidate the regulation of CCL20 secretion by BEC, we measured CCL20 mRNA and protein secretion from human BEC in response to cytokine treatment. *CCL20* mRNA was detected in untreated BEC and increased markedly in response to cytokine treatment (Supplementary Fig. 1A) accompanied by an increase in secreted CCL20 in response to IL-1β, TNF-α + IFN-γ, and IL-17 (Fig. 3B).

### Peri-ductal Th17 positioning via CCL20-CCR6 and CXCL9–11 CXCR3

To determine whether BEC-derived chemokines attract Th17 cells, we studied the migration of Th17 cells in chemotaxis experiments to BEC-conditioned media (Fig. 3C). Th17 expressing CCR6 and CXCR3 migrated towards conditioned media from BEC stimulated with IL-17 (Chemotatic index 2.5 times control). This migration was significantly reduced by blocking CCL20 and the CXCR3 ligands CXCL9–11 or by treating Th17 cells with anti-CXCR3 (Fig. 3C). Thus, IL-17 stimulates CCL20 and CXCL9–11 expression by BEC leading to the recruitment of CCR6^+^ CXCR3^+^ Th17 to bile ducts. Th17 cells may then establish a positive feedback loop by amplifying secretion of CCL20 by activating the IL-17 receptor on BEC.

### Th17/Tc17 adhesion to HSEC under flow is dependent on CXCR3, ICAM-1, and VCAM-1

**Supplementary Fig. 2:**
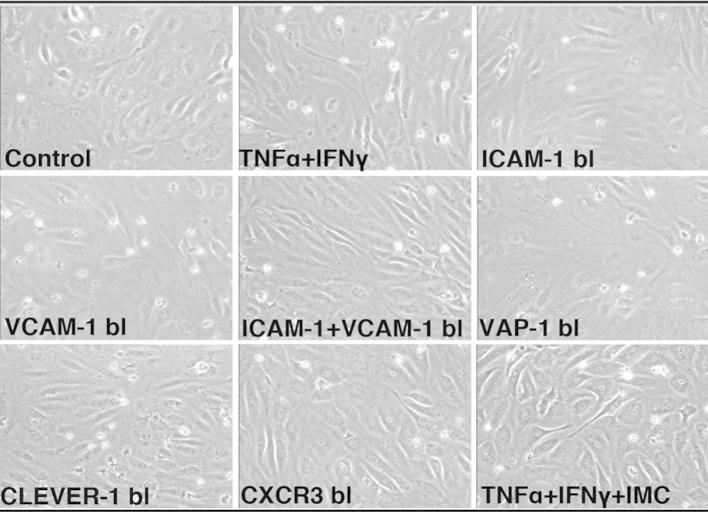


We used HSECs treated with IFN-γ and TNF-α in flow-based adhesion assays to model inflamed HSEC, which express ICAM-1, VCAM-1, and CXCR3 ligands in chronic hepatitis [25]. Both Tc17 (Fig. 3D) and Th17 (Fig. 3E) adhered to IFN-γ and TNF-α-stimulated HSEC under flow and when flowed over cytokine-stimulated HSECs, Th17/Tc17 displayed brief rolling/tethering interactions followed by arrest and stable adhesion (Supplementary Fig. 2). Cells undergoing stable adhesion from flow were significantly reduced by treating T cells with anti-CXCR3 or by endothelial treatment with anti-ICAM-1 and anti-VCAM-1 (Supplementary Figs. 2 and 3F, G). CLEVER-1, which is involved in regulatory T cells recruitment, had no impact on Th17/Tc17 recruitment (Fig. 3F and G). All experiments were compared with control microslides, in which control antibodies were used.

### Recruitment of Th17 cells in response to acute and chronic liver injury in mice

**Fig. 4:**
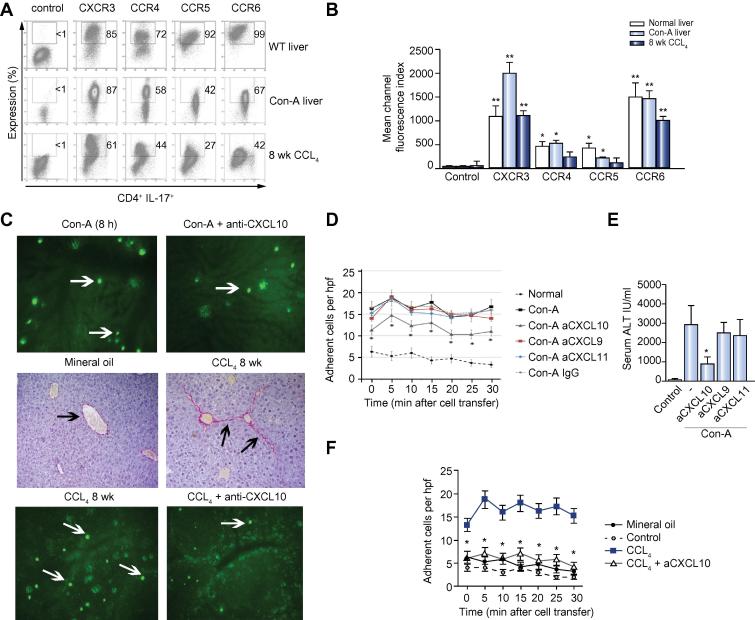
**Chemokine-mediated Th17 recruitment to the injured murine liver**. (A and B) Chemokine receptor expression by flow cytometry on liver-derived Th17 cells from ConA-induced acute hepatitis and CCL_4_-induced chronic liver injury (mean ± SEM. N = 6; ^∗^*p *<0.05; ^∗∗^*p *<0.01; Paired *t* test). (C, D and F) *Ex-vivo* generated, CFSE-labelled Th17 were injected (C and D) 8 h after ConA or (C and F) 1 week after week 8 CCL_4_ injection, and recruitment investigated by IVM. Still images of treatment adherent cells in hepatic sinusoids after 30 min are shown (C, D and F). The graph shows the mean number of adherent cells from six high-power fields ± SEM at each time point with at least 3 animals/group for (D) ConA and (F) CCL_4_. Data are compared to control animals that received saline injections. Anti-CXCL9–11 or control antibodies were injected 1 h after (D) ConA injection and (F) anti-CXCL10 or control antibodies for CCL_4_. (C, middle panel) CCL_4_ liver fibrosis was confirmed by Sirius Red staining (black arrows). (E) Serum ALT levels after ConA injection comparing blocking CXCL9–11. (Data represents mean ± SEM. N = 6; ^∗^*p *<0.05; paired *t* test.) [This figure appears in colour on the web.]

We investigated the role of CXCR3 in Th17 recruitment *in vivo* in two mouse models of liver inflammation. C57Bl6 mice developed a severe acute hepatitis 8 h after tail vein injection with Con A, and C57Bl6 mice, treated for 6 weeks with biweekly intra-peritoneal CCL_4_, develop chronic liver injury, inflammation, and fibrosis. Th17 cells isolated from livers of Con-A or CCL_4_-treated mice expressed high levels of CXCR3 and CCR6 comparable to their human equivalents (Fig. 4A and B). Intermediate levels of CCR4 and CCR5 were also detected and decreased as the liver injury became chronic.

IVM (Supplementary Videos 1–4) was used to study the recruitment of adoptively transferred Th17 cells via the hepatic sinusoids into the inflamed murine liver. CFSE-labelled *ex-vivo* generated Th17 cells were infused intra-arterially and their migratory behaviour and adhesion to hepatic sinusoids (Fig. 4C, D and F) and hepatic injury (Fig. 4E) was recorded in animals with liver inflammation/injury and in controls (Fig. 4C and D). Significantly more Th17 cells adhered to the sinusoidal endothelium in both injury models compared with control mice, and blocking CXCR3 ligand inhibited this adhesion (Fig. 4C, D and F). In the Con-A model, anti-CXCL10 only partially inhibited adhesion whereas in CCL_4_-treated animals it reduced adhesion to the levels observed in control animals (Fig. 4C, D and F) (Supplementary Videos 1–4).

## Discussion

Th17 cells are implicated in chronic inflammatory liver diseases and hepatocellular carcinoma. We detected IL-17 secreting cells in several liver diseases. IL-17^+^ cells constituted 2–3% of CD3^+^ infiltrate and included both Th17 and Tc17 cells. IL-17^+^ cells were detected in both lobules and portal tracts and concentrated around bile ducts. The frequencies were increased in all diseases studied. We investigated the molecular mechanisms responsible for their recruitment and positioning within the liver.

Chemokine receptors play a critical role in the recruitment of T cells to tissue and the selective expression of chemokine receptors on subsets of T cells together with the restricted expression of their chemokine ligands in tissues determines which particular T cell subsets are recruited to organs or sites of inflammation. In order to determine the chemokine receptors involved in Th17/Tc17 cell recruitment to the human liver, we analysed expression of chemokine receptors on these cells. Intra-hepatic Th17 and Tc17 cells expressed high levels of CXCR3 and CCR6, and Tc17 cells also expressed CXCR6 at high levels.

We previously reported a critical role for CXCR3 in the recruitment of regulatory T cells in response to CXCL9–11 secreted by parenchymal cells and posted on the luminal surface of HSEC via interactions with proteoglycans in the glycocalyx [25], [27]. CXCL10 expressed by stromal cells subsequently supported migration of CXCR3^+^ T cells into the hepatic parenchyma or along myofibroblast conduits in the space of Disse to the portal tracts and biliary epithelium [25], [28], [31]. We now show that Th17 and Tc17 cells migrate to CXCR3 ligands *in vitro* and use CXCR3-mediated signals to bind to the sinusoidal endothelium from flow. We confirmed this *in vivo* in two different types of experimental liver injury. In the *in vitro* chemotaxis assays, we saw reduced migration after depleting the three CXCR3 ligands individually, and a greater effect when blocking CXCR3. These data show that all three chemokines are present in the supernatants and able to activate CXCR3. The ability to inhibit responses by depleting only one of the three ligands would suggest non-redundant roles for the three chemokines, a surprising finding in a simple migration assay. However, the analysis is complicated by the fact that CXCL10 and CXCL11 are allosteric ligands for CXCR3; CXCL10 and CXCL9 have vastly different affinities for uncoupled CXCR3 when compared with CXCL11, and CXCL10 and CXCL11 are allotopic ligands for coupled CXCR3 [29], [30]. Thus, the functional outcome of interactions between ligands and receptor is complex. This complexity may contribute to the more marked effect of blocking CXCL10 *in vivo* on liver injury but here the situation is further complicated by differential expression of the three ligands in response to injury.

Thus, we have shown for the first time that Th17 cells use CXCR3-dependent pathways to enter the liver. These findings suggest that CXCR3 is the dominant receptor in promoting lymphocyte recruitment into the inflamed liver. The selectivity of recruitment does not occur at the level of the endothelium but subsequent signals determine where subsets of cells migrate within the inflamed liver. CXCR3 signals alone may be sufficient to recruit Th17 to the hepatic lobules but it is likely that other signals are required to position Th17 cells in portal tracts. The relatively high numbers of Th17 cells, which we and others have observed around bile ducts [19], led us to investigate factors that attract Th17 to inflamed bile ducts.

Cholangiocytes secrete an array of chemokines in response to inflammation. We had previously reported CD8 effector and regulatory T cells use CXCR6 and CCR10 respectively, to localise at periductal area in portal tracts, and the present study suggests that CCR6 plays this role for Th17 cells [24], [31]. We detected high levels of CCR6 on intrahepatic Th17 cells, but this receptor was not involved in recruitment via sinusoids. This led us to determine where its ligand CCL20 is expressed in the liver and we found it to be largely restricted to bile ducts. Furthermore, cholangiocytes express CCL20 and IL-17RA and also secrete CCL20 in response to proinflammatory cytokines including IL-17. Th17 cells showed CCL20-dependent migration to cholangiocytes suggesting this pathway could be important in the positioning of Th17 cells in portal tracts in liver diseases. The fact that IL-17 secreted by Th17 cells increases CCL20 expression from cholangiocytes, creates a positive feedback loop through which Th17 cells can amplify the recruitment of more IL-17-secreting effector cells to the bile ducts. Th17 are important in controlling bacterial, fungal, and other pathogens, which might enter the liver from the gut via the biliary epithelium [8], [32] and requirement to provide protection at this epithelial interface may explain why Th17 are positioned at this site.

We have shown in this study that some intrahepatic IL-17 cells also secrete IL-22 that is important for epithelial healing and repair [21], [33]. The role of IL-17 and Th17 in liver injury is unclear and little is known about the balance of damaging inflammatory effects and beneficial reparative responses driven by IL-17 or Th17 cells in the liver. Studies in animal models provide conflicting data. Con-A hepatitis is ameliorated in IL-17 and IL-17 receptor knockout animals suggesting that IL-17 is pro-inflammatory in this model [34]. However, Lafdil *et al.* reported that although there was a reduction in hepatitis severity in *IL-17*^−/−^ animals, most of the hepatitis was driven by IFN-γ, because *IFN-γ*^−/−^ animals were protected from liver injury [35]. Zenewicz *et al.* compared the effects of Con-A hepatitis in wild type, *IL-22*^−/−^, *IL-17*^−/−^, and *IL-17/22* double knock-out mice and reported that IL-22 and, to a lesser extent IL-17, provided relative protection from Con-A-induced liver injury [21]. A possible explanation for these findings is that IL-17 from multiple cellular sources can influence liver inflammation at several levels and the precise role of IL-17 will depend on the timing, duration, and nature of the inflammation. We conclude that Th17 cells use CXCR3 to enter the liver but require further signals via CCL20-CCR6 to migrate to the portal tracts and localise near bile duct that express the IL-17 receptor, where they are ideally located to provide protection against pathogens entering the liver [36].

## Financial support

Medical Research Council (MRC) UK, University Hospitals Birmingham (UHB) Charity, National Institute for Health Research (NIHR).

Ye Htun Oo was supported by Medical Research Council Intermediate Clinical Fellowship and Vanessa Banz by an EASL Sheila Sherlock Fellowship.

This research was supported by the National Institute for Health Research (NIHR) Birmingham Liver Biomedical Research Unit based at University Hospital Birmingham NHS Foundation Trust and the University of Birmingham. The views expressed are those of the author(s) and not necessarily those of the NHS, the NIHR or the Department of Health.

Paul Klenerman ackowledges NIHR Biomedical Research Centre (oxford), Wellcome Trust (WT091663MA), and NIAID U19 Bio-defense Program (NIH NIAID 1U19AI082630-01).

## Conflict of interest

The authors who have taken part in this study declared that they do not have anything to disclose regarding funding or conflict of interest with respect to this manuscript.

## Appendix A Supplementary data

Supplementary Data This document contains Supplementary methods and figure captions.

Supplementary video 1 Invtrvital microscopy of murine Th17 cells recruitment via hepatic sinusoids in CCL4 injury model.

Supplementary video 2 Invtrvital microscopy of murine Th17 cells recruitment via hepatic sinusoids with IP-10 block in CCL4 injury model.

Supplementary video 3 Invtrvital microscopy of murine Th17 cells recruitment via hepatic sinusoids in ConA injury model.

Supplementary video 4 Invtrvital microscopy of murine Th17 cells recruitment via hepatic sinusoids with IP-10 block in ConA injury model.
