# Supplementary material for: CXCR3-dependent recruitment and CCR6-mediated positioning of Th-17 cells in the inflamed liver
Source: J Hepatol. 2012 Nov;57(5):1044–51. doi: 10.1016/j.jhep.2012.07.008 (PMC3994510; doi:10.1016/j.jhep.2012.07.008)
Supplement: Supplementary Data — This document contains Supplementary methods and figure captions. [file mmc1.docx]

**METHODS:**

**Mice and Human tissue**.

Male wild type C57BL/6 mice (6-8 weeks) were obtained from existing colonies at the University of Birmingham. Mice were bred and maintained under controlled animal care conditions. All procedures were carried out after ethical and United Kingdom Home Office approval in accordance with the Animals (Scientific Procedures) Act of 1986.

Venous blood was obtained from patients undergoing liver transplantation before induction of anaesthesia. Subsequently explanted diseased liver was obtained from the same patients to allow the isolation of matched blood and liver-derived cells. The following patient groups were studied primary biliary cirrhosis (PBC), auto-immune hepatitis (AIH), chronic hepatitis C virus infection (HCV), alcoholic liver cirrhosis (ALD), non-alcoholic steatohepatitis (NASH). The final diagnosis was based on characteristic clinical, biochemical, immunological and histological findings. Non-diseased liver tissue was obtained from surplus donor liver tissue or from uninvolved liver tissue removed at the time of resection for colorectal hepatic metastases. All samples were collected with informed patient consent and local research ethics committee approval (local research ethics committee approval 04/Q2708/41 and REC 2003/242).

**Antibodies and reagents**

The following antibodies were used for multi-colour flow cytometry: mouse anti-human CD4 Pacific Blue (RPA-T4, BD Pharmingen ), mouse anti-human CD3 PE-Cy7 (UCHT-1, eBioscience), mouse anti-human CD8 mAb PECy5.5 (clone3B5), CCR4 mAb (205410 /FAB 1567P, FAB1567F, R&D Systems), CCR6 mAb (53103/FAB 195P), CXCR3 mAb (FAB160P, FAB160F), CXCR6 mAb (56811/FAB699P) all from R&D Systems. For intracellular cytokine staining, FITC conjugated mAb IFN-γ (340449, BD Pharmingen), FITC conjugated mAb IFN-γ (MHCIFG05, Caltag), FITC conjugated mAb to TNF-α (554512, BD Pharmingen), PE conjugated IL-17 mAb (eBioscience; eBio64CAP17), APC conjugated IL-22 mAb (eBioscience; 22URTI), anti-Human/Mouse ROR gamma(t) PE (AFKJS-9) IL-17 RA APC conjugated antibody (eBioscience; 424LTS), Anti-cytokeratin19 antibody (RCK108; ab9221).

**Isolation of human liver infiltrating lymphocytes**

Fresh isolation of human liver infiltrating lymphocytes from various diseased explanted livers was carried out for phenotyping. Briefly, resected liver tissue was reduced to 5-mm^3^ cubes and placed for 5 minutes at 230rpm in a Stomacher 400 circulator (Seward, UK). Mechanical digestion was applied to preserve chemokine receptors and resultant homogenized tissue was filtered through a fine gauze mesh and lymphocytes were separated by layering and over a Lympholyte® density gradient (Cedarlane) and centrifugation at 650 X *g* for 30 minutes. Isolated fresh liver infiltrating lymphocytes were phenotyped on the day of isolation without expansion or activation.

**Isolation and culture of biliary epithelial cells (BEC).**

Biliary epithelial cells were isolated in our laboratory according to established protocols. Briefly, the cells were purified by density gradient centrifugation above 25% metrizamide (Nycomed) at 450g for 20 minutes. BEC were then isolated by positive magnetic selection using an antibody raised against the epithelial specific cell-surface glycoprotein HEA-125 (10µg/ml, Progen). BEC cell phenotype was confirmed by high-level expression of cytokeratin 19. Cells were cultured to confluence and BEC media was changed every other day or as required. Cells were used between passage 2-4 for ELISA and chemotaxis experiments.

**Isolation and purification of Th17 cells**

Spleens (or livers) were removed and prepared for single cell suspensions using gentleMACS (Miltenyi Biotec). After filtering through a mesh, the cell suspension was layered on a Lympholyte® density gradient (Cedarlane) and centrifuged for 20 minutes at 2000rpm. Further isolation of CD4+ T cells was carried out using the MACS magnetic bead column separation kit according to the manufacturers protocol (Miltenyi Biotec). Isolation of IL-17 secreting cells from human blood was carried out according to the manufacturer’s protocol using the IL-17 Cell Enrichment and Detection Kit (Miltenyi Biotec). CD4+ T-cell fraction was always greater than 95% and cell viability was checked using tryphan blue.

**Th17 culture conditions**

CD4+ selected murine splenocytes or human PBL (purity >95%) were stimulated with anti-CD3/CD28 beads in Iscove's Modified Dulbecco's Medium (IMDM) supplemented with 10% (vol/vol) heat-inactivated FCS (all Invitrogen), benzylpenicillin/streptomycin (100IU/ml), glutamine (2mM) (all from Sigma Aldrich). The culture medium was supplemented with specific recombinant cytokines and anti-cytokine mAbs as follows for Th17 differentiation:

*Murine conditions*: TGFβ1 (2ng/ml, R&D Systems), rIL-6 (50ng/ml, Pepro Tech), anti-IL-4 (10ug/ml, BD Biosciences, clone 11B11), anti-IFNγ (10ug/ml, Novus Biologicals clone XMG1.2). Cells were split 1:2 after 24-48h and the medium supplemented with the previously listed cytokines and anti-cytokine mAbs as well as rIL-23 (50ng/ml, R&D Systems) and rIL-2 (Sigma Aldrich, 200IU/ well). Optimal differentiation was reached after one week in culture. Control CD4+ cells were stimulated with anti-CD3/CD28 beads and cultured in complete IMDM supplemented with rIL-2.

*Human conditions*: human IL-1β (50ng/ml) R&D, IL-6 (20ng/ml), IL-2 (40IU/ml), IL-23 (100ng/ml), IL-21 (50ng/ml), anti-IL-4 (500ng/ml) and anti-IFNγ (5ug/ml, all from R&D Systems) also cultured in IMDM media enriched with AhR ligands supplemented with fetal calf serum and rIL-2. Optimal differentiation was reached after two weeks in culture and purity was confirmed by flow cytometry.

**ELISA**

Chemokines were measured in culture supernatants from human biliary epithelial cells stimulated with IL-17A (50ng/ml), TNF-α (10ng/ml), IFN-γ(100ng/ml), IL-1-β(50ng/ml) or unstimulated cells by sandwich ELISA using Quantikine Human Immunoassay kits specific for human CCL20 (DM3A00) (R&D Systems). All measurements were performed using triplicate samples for each experiment.

**Single and dual colour immunohistochemistry**

Staining was carried out on paraffin-embedded human liver sections using goat polyclonal anti-human IL-17A Ab (Clone AF 317-NA, 0.75µg/ml, R&D Systems) optimized on tonsil sections. Sections were deparaffinized, rehydrated, endogenous peroxidase blocked and antigen retrieval carried out. Non-specific binding was blocked with diluted serum (rabbit IgG ABC kit; Vector stain, Vector Laboratories) and sections incubated with normal goat IgG as a negative control. Peroxidase activity was visualized using the 3,3'-Diaminobenzidine (DAB) and sections counter-stained with haematoxylin. Dual immunostaining with IL-17A and anti-HEA for biliary epithelial cells (61004, PROGEN) was visualised using Vector NovaRed (Vector Laboratories) and sections counterstained in haematoxylin.

**Confocal microscopy**

Paraffin-embedded human liver tissue sections were deparaffinised, then rehydrated and antigen retrieval done in EDTA buffer. Slides were then incubated in horse serum and goat polyclonal IL17 (1:100; 1 µg/ml, AF317-NA, R&D systems) followed by incubation in donkey-anti-goat FITC (Jackson Lab) and rabbit-anti-FITC (Jackson Lab) followed by goat anti-rabbit (Jackson Lab). Images were taken with a Zeiss confocal microscope. Mouse anti-human ROR gamma(t) purified (eBioscience; AFKJS-9), CD3 (eBioscience; UCHT1), Foxp3 (Abcam; ab20034) were used as primary and the following secondary and tertiary Abs were used for confocal microscopy. Anti-IgG1 FITC (Southern Biotech), Rabbit-anti FITC (Sigma), Goat-anti Rabbit FITC (Southern Biotech), Anti-IgG2b biotinylated (Southern Biotech), Anti-IgG1 biotinylated (Southern Biotech), Streptavidin 555 (Invitrogen); Streptavidin 647 (Invitrogen), Mouse Anti-human IgG2a Cy-3 (Southern Biotech), goat anti-mouse IgG1 FITC (Southern Biotech).

**Flow cytometry**

All data was collected using a nine colour Dako Cyan flow cytometer and analyzed with Summit 4.3 software (Dako Cytomation). Freshly isolated liver infiltrating lymphocytes from different explanted diseased livers were used. For intracellular staining, cells were stimulated with 100ng/ml of PMA and 1 µg/ml of ionomycin for 5 hours and incubated with 4µg/ml of Brefeldin A for the last 3 hours of stimulation. For intracellular staining of IL17, IL-22 IFN-γ and RORc liver infiltrating lymphocytes were fixed and permeabilized with staining buffer (eBioscience) according to the manufacturer’s protocol. 1x10^6^ cells stained with an anti-mouse-IL17 mAb, IL-22mAb directly conjugated to PE and APC and IFN-γ mAb conjugated to FITC, or RORγt conjugated to PE (eBioscience).

**Semi-quantitative PCR**

RT PCR was performed in order to examine the presence of CCL20, and IL-17RA from both unstimulated and biliary epithelial cells (BEC) stimulated with different cytokines ; 17A (50ng/ml), TNF-α (10ng/ml), IFN-γ(100ng/ml), IL-1-β(100ng/ml). Primers were designed from GenBank sequences for human CCL20 and GAPDH : CCL20 forward 5'-GCGCAAATCCAAAACAG CT-3' ; CCL20 reverse 5'-CAAGTCCAGTGAGGCACAAA-3'; IL-17RA forward 5’-CCA GAT CCC AGC TTT GAG AG-3’; IL-17RA reverse 5’-AAA TGC CCG CCA CAT AGT AG-3’. Positive and negative controls (no cDNA) were included in each assay. Product size for CCL20=200bp; IL-17RA=285bp. Density of messenger RNA was then compared between unstimulated BEC with different cytokines stimulated BECs.

**Th 17 cell Chemotaxis**

Transwell chemotaxis was assessed using 5µ pore, gelatin-coated chemotaxis inserts (Corning). Primary BEC cultures were stimulated with IL-17A or left in culture medium for 24hr when supernatants were collected, placed in the bottom chamber with isolated autologous Th17 cells in the upper chamber. Anti-CCL20 mAb (67310; 5μg/ml; R&D Systems), anti-CXCL9 mAb (49106; 50μg/ml; R&D Systems), anti-CXCL10 mAb (33036/ 5μg/ml; R&D Systems) or anti-CXCL11 mAb (87328; 5μg/ml; R&D Systems) antibody were added to selected lower chambers. Anti-CXCR3 chemokine receptor block (49801; 10μg/ml; R&D Systems) was applied 30min before the experiments on Th17 cells and then added to upper chambers. Data are expressed as fold reduction in migrated cells comparing IL-17 stimulated BEC supernatant media with individuals block in assays. Experiments were done in triplicate.

**Flow based adhesion assay**

The recruitment of Th17/Tc17 was studied by using flow based adhesion assays. HSEC were cultured in microcapillaries, stimulated with TNF-α (10ng/ml) and IFN-γ (10ng/ml) as previously described. Total adhesion was calculated as cells/mm2 normalised to the number of Th17/Tc17 cells perfused. In function-blocking experiments HSEC were pre-treated with antibodies against ICAM-1 (10μg/ml; R&D), VCAM-1 (10μg/ml; Millipore), VAP-1 (TK8-14, 10μg/ml; Biotie, Finland), CLEVER-1 (3-702, 10μg/ml; Finland) and cells were pretreated with anti-CXCR3 (10μg/ml; R&D). IgG1 and IgG2b (10μg/ml; Dako&eBioscience) were used as negative control.

**Murine liver injury models**

Induction of Concanavalin A (ConA) mediated hepatitis. Concanavalin A (ConA) induced hepatitis is widely used as an animal model of T cell-mediated hepatitis. ConA V (Sigma Aldrich) was dissolved in sterile PBS to a concentration of 13.7mg/kg bodyweight. Mice received a single intravenous dose of ConA via tail vein injection and were sacrificed 8-10 hours post-injection. Anti-CXCL10 antibodies (50ug/mouse; clone 134013, R&D Systems) or isotype control antibodies added 1 hour after Con A injection.

Carbon tetrachloride (CCL4). Administration of carbon tetrachloride (CCL4) to rodents causes hepatocyte injury characterized by centrilobular necrosis followed by fibrosis. CCL4 was administered bi-weekly by intraperitoneal injection (1:3 dissolved in mineral oil, total of 40ul/mouse). Control animals received mineral oil only. Mice received either CCL4 or mineral oil only over a period of 8 consecutive weeks

**Intra-vital microscopy**

Anaesthesia was induced with an intra-peritoneal injection of ketamine (100 mg/kg Vetalar; Pharmacia and Upjohn Ltd, UK) and 2% xylazine (10 mg/kg; Millpledge Pharamaceuticals, UK). Cannulae were inserted in the trachea to facilitate spontaneous respiration and in the left carotid artery to provide a route for administration of additional anesthesia and access for administration of fluorescently labelled cells. Midline laparotomy was followed by careful mobilization of the left liver lobe. Body temperature was maintained at 37°C during the entire experiment. Animals were transferred to the stage of a motorized Olympus BX-61WI microscope equipped with a water immersion objective (Olympus UmplanFI 10x/0.30w). A field of view for analysis was pre-selected. Following this, previously in vitro generated Th17 cells were labelled with 5 µM CFSE (Molecular Probes, Invitrogen, Paisley, UK) and introduced systematically. 5x106 CFSE labelled cells were injected / experiment. Cells were defined as adherent if they remained stationary in the field of view for > 30 seconds, with the other cells classified as free flowing. Figures represent the mean adhesion (or free flowing cells) ± SEM of at least 3 animals per group. In order to ensure that the initial field chosen for analysis was representative of the whole liver, six further fields of view were chosen at random and analyzed at the end of the viewing period. Digital images were collected using a high capture rate Sensicam CCD camera (the Cooke Corporation, USA). A high performance GEN III image intensifier (Videoscope Int. Ltd., USA) incorporated between the microscope and camera amplified light 1000-fold, allowing for visualization of low fluorescent intensities. The images were collected using Slidebook software (Intelligent Imaging Innovations, USA) and stored as permanent digital images for off-line analysis.

**Supplementary Figure 1.**

**Positioning of IL-17 cells around bile ducts and IL-17RA expression on biliary epithelial cells**

A) CCL20 and IL-17 receptor mRNA in unstimulated and different cytokines stimulated BEC is demonstrated by RT-PCR, GAPDH was used as control gene. Quantification of mRNA density of unstimulated biliary epithelial cells (normalised as 1) was compared with mRNA density of different cytokine stimulated biliary epithelial cells was performed. The analysis of three BEC sample was shown (Oneway ANOVA test *p<0.01; **p<0.001; ***p<0.0001. B) IL17+ cells are located around bile ducts which were shown by immunohistochemistry. 6 disease livers were stained with IL-17 antibody (representative immunohistochemistry of primary biliary cirrhosis was shown). IL-17 receptor (IL-17RA) expression on BEC was investigated and shown by flow cytometry overlay histogram. (IL17 receptor expression on BEC cells in un-stimulated condition =grey or TNF-α and IFN-γ stimulated condition=black). BEC cells purity was confirmed by their expression of CK19 (black) or control antibody (white) shown in overlay histogram. One overlay flow cytometry histogram of 4 experiments was shown; Mean±SEM. C) LITh17&LITc17 are confined to CD161^high^ population. Liver infiltrating lymphocytes are gated on forward and side scatters and this gating was applied to CD3 vs CD4 or CD3 vs CD8 population and this gating was applied to either control antibody or IL-17 antibody vs CD161. One representative flow cytometry blot (HCV) of six experiments was shown; Mean±SEM; (2 x chronic hepatitis C (HCV), 1 x alcoholic liver disease, 2 x primary biliary cirrhosis, 1 x autoimmune hepatitis). LITh17 (gated on LITh17 population and LITc17 (gated on LITc17) cells expressed high level of transcription factor, RORC (One representative flow cytometry blot of N=5). **Supplementary Figure 2**

Video images of flow based adhesion assays on human HSECs treated with IFN-γ and TNF-α to model inflamed HSEC, which expresses ICAM-1, VCAM-1, VAP-1 and CXCR3 ligands in chronic hepatitis. When flowed over cytokine-stimulated HSECs, both Th17/Tc17 displayed brief rolling/tethering interactions followed by arrest, stable adhesion. The numbers of cells undergoing stable adhesion from flow was reduced by Th17/Tc17 treatment with anti-CXCR3 or endothelial treatment with antibodies against VCAM-1, ICAM-1, VAP-1 or Th17/Tc17 treated with anti-CXCR3. CLEVER-1 had no impact on Th17/Tc17 recruitment. All experiments were compared with control microslides in which control antibodies were used. One representative experiment video image was shown.

**Intravital microscopy video1-4**
